# Analysis of Clinical Characteristics, Radiological Predictors, Pathological Features, and Perioperative Outcomes Associated with Perinephric Fat Adhesion Degree

**DOI:** 10.1155/2021/9095469

**Published:** 2021-12-27

**Authors:** Junqiang Liu, Yiheng Jiang, Hongwei Huang, Zheng Zhu, Jing Chen, Dikuan Liu, Lina Wang, Huafeng Zong, Xishuang Song, Xuejian Wang, Xinqing Zhu, Jianbo Wang, Sony Nahayo, Qiwei Chen, Deyong Yang

**Affiliations:** ^1^Department of Urology, First Affiliated Hospital of Dalian Medical University, Dalian 116021, China; ^2^Department of Internal Medicine, University of California, Davis, USA; ^3^Department of Imaging, First Affiliated Hospital of Dalian Medical University, Dalian 116021, China; ^4^Department of Pathology, Dalian Friendship Hospital, Dalian 116021, China; ^5^School of Information Science and Technology of Dalian Maritime University, 116000 Dalian City, Liaoning Province, China

## Abstract

**Background:**

To assess the clinical characteristics, radiological predictors, and pathological features of perinephric fat adhesion degree (PFAD) graded based on fixed criteria and to determine the impact of adherent perinephric fat (APF) on retroperitoneal laparoscopic partial nephrectomy (RLPN) outcomes.

**Methods:**

84 patients undergoing RLPN were included and graded into 4 groups based on PFAD. Univariate and multivariate analyses were performed for clinical characteristics and radiological predictors of PFAD. Perioperative data were compared between APF groups and non-APF groups. Masson staining determined collagen fibers. Immunohistochemistry detected CD45 immune cells and CD34 vessels.

**Results:**

20, 28, 18, and 18 patients were graded as normal perinephric fat (NPF), mild adherent perinephric fat (MiPF), moderate adherent perinephric fat (MoPF), and severe adherent perinephric fat (SPF), respectively. Multivariate analysis revealed that gender (*p* < 0.001), age (*p* = 0.003), and hypertension (*p* = 0.006) were significant clinical risk factors of PFAD, while radiological predictors included perinephric stranding (*p* = 0.001), posterior perinephric fat thickness (*p* = 0.009), and perinephric fat density (*p* = 0.02). APF was associated with drain output (*p* = 0.012) and accompanied by immune cells gathering in renal cortex near thickened renal capsule with many vessels.

**Conclusions:**

Clinical characteristics and radiological predictors can evaluate PFAD and may assist to guide preoperative surgical option. Pathological features of APF reflect decapsulation and bleeding during kidney mobilization at RLPN.

## 1. Introduction

For T1 stage renal cell carcinoma, especially for T1a tumors, partial nephrectomy is recognized as the standard operation whenever technically feasible [1–3]. In RLPN, multiple radiological scores that may anticipate tumor complexity are proposed to describe the tumor-specific characteristics, but they neglect other factors. One of the potential patient-specific factors for surgical complexity is APF characterized by thickening and adhesion fat surrounding the kidneys [4].

Due to lack of consensus objective grading criteria for the definition of APF, the incidence of APF varies greatly, ranging from 10.6% to 55.2% according to the previous studies [4, 5]. Most researchers conducted retrospective analysis based solely on the description of APF adhesion in surgical records [6–8]. Zheng et al. [9] graded APF according to the operative time for renal mobilization. Dariane et al. [10] graded APF based on the subjective surgical score for decapsulation during dissection. Despite these attempts to improve objectivity, there appears to be no clear criteria to grade PFAD.

So far, the pathogenesis of APF remains unclear. At present, the mainstream argues that APF has a positive correlation with chronic inflammation. Previous studies showed interleukins and adipocyte size were increased in APF [10, 11]. But these findings failed to reflect the clinical, radiological, and surgical characteristics of APF.

Therefore, our study established objective criteria after multiple surgical videos were reviewed. The present study is to prospectively assess patients' clinical characteristics and radiological predictors for PFAD graded according to objective criteria and to analyze the impact of APF on RLPN outcomes and pathological features of APF. Moreover, we explored the relationship between clinical characteristics, imaging findings, and pathology of APF.

## 2. Methods

### 2.1. Patients and Data Collection

We performed a prospective study of patients who underwent RLPN for suspicious renal mass between March 2017 and March 2018 at the First Affiliated Hospital of Dalian Medical University. The study was conducted according to the guidelines of the Declaration of Helsinki after approval from our hospital's institutional ethical committee (First Affiliated Hospital of Dalian Medical University). The clinical variables (age, gender, body mass index (BMI), preoperative eGFR, hypertension, diabetes, active smoking, and alcoholism), perioperative outcomes (operative time (OT), warm ischemia time (WIT), estimated blood loss (EBL), transfusions, Clavien–Dindo classification (CDC), length of stay, drain output, postoperative gastrointestinal recovery time, postoperative fever, gastrointestinal discomfort, and change dressing frequency), and postoperative pathology results (Fuhrman grade) were collected.

### 2.2. Criteria for PFAD Grading

**Figure 1:**
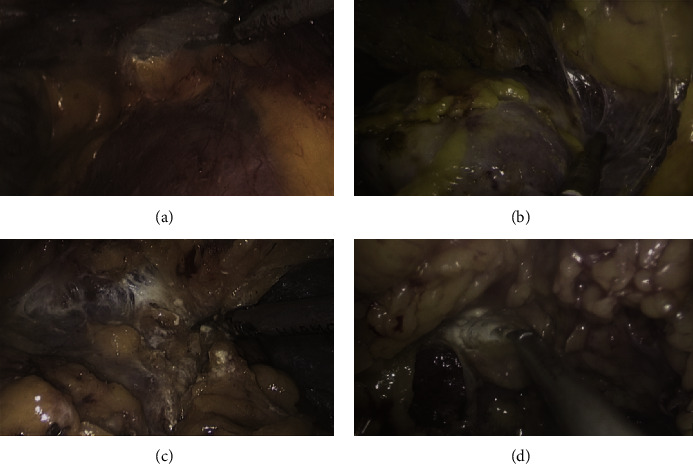
Surgical criterion of APF. (a) Normal perinephric fat (NPF). (b) Mild adherent perinephric fat (MiPF). (c) Moderate adherent perinephric fat (MoPF). (d) Severe adherent perinephric fat (SPF).

PFAD is divided into four grades (Figure 1). Normal perinephric fat (NPF): easily blunt dissect perinephric fat from kidney, less fibers connect renal capsule and fat; mild adherent perinephric fat (MiPF): easily blunt dissect, more fibers connect renal capsule and fat, and scattered flaky fat remains on the renal capsule after blunt dissection; moderate adherent perinephric fat (MoPF): part of perinephric fat adheres to the kidney and need sharp dissection; and severe adherent perinephric fat (SPF): all parts of perinephric fat adhere to the kidney and need sharp dissection or subcapsular dissection. NPF and MiPF were defined as non-APF, which did not complicate kidney mobilization while the latter two grades were APF.

### 2.3. Radiological Data

**Figure 2:**
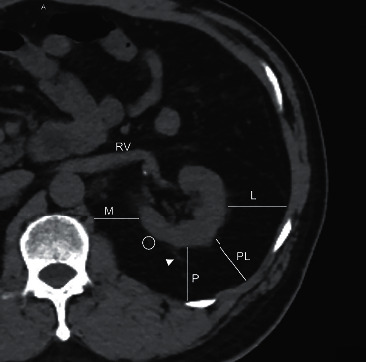
Measurement of perinephric fat at the level of the vein. M, medial perinephric fat thickness; L, lateral perinephric fat thickness; P, posterior perinephric fat thickness; PL, posterolateral perinephric fat thickness; circle, HU of perinephric fat; triangle, stranding; RV, renal vein.

All patients underwent preoperative abdominal computed tomographic (CT) imaging. The thickness of the medial, lateral, posterior, and poster lateral perinephric fat were measured at the level of the renal vena cava according to the method described by Eisner et al. [12]. Perinephric fat density was a manually selected area close to the renal capsule, and Hounsfield units (HU) in this area were calculated automatically. Perinephric fat area was defined as the area between the medial and posterior renal fat thickness measurement line. Perinephric stranding was determined as Kim et al. [13] described (Figure 2).

The preoperative CT was assessed by two urologists (J.L. and H.H.) blinded to patient PFAD status independently. If there were any differences between the two observers, the corresponding image was assigned to another highly qualified urologist (D.Y.) for final results.

### 2.4. Histological Analysis

The isolated perinephric fat and adjacent kidney were taken from the NPF and the SPF, fixed in 4% formalin buffer, embedded in paraffin, and cut into 4 mm thick serial sections. The slices were stained by the Masson method. The primary antibodies against CD45 (1 : 200) and CD34 (1 : 200) were from Proteintech (China). After staining, the samples were observed under the microscope. Light yellow, brown yellow, or dark brown was supposed as the positive expression. Positive control for Masson staining was defined as renal capsule stained. Positive control for CD34 was defined as blood vessels stained (brown in the cytoplasm). Positive control for CD45 was defined as renal cortex or renal capsule stained.

Besides, an additional file shows the immunodetection methods (Additional file 1). We performed immunohistological staining of CD34, a marker of endothelium (positive in glomerular while negative in kidney tubules), and CD45, a marker of immune cells (positive in lymph nodes while negative in normal kidney tissues) in this research.

### 2.5. Statistical Analysis

Continuous variables were reported as median (minimum and maximum) and qualitative variables as ratio (percentage). Univariate and ordered multivariate logistic regression analyses were performed to assess clinical characteristics and radiological predictors of PFAD. The chi-square test was used to compare categorical data, and the Mann–Whitney U test was used to compare nonnormally distributed continuous variables and ordered qualitative variables for perioperative outcomes or pathological grading of renal cell carcinoma (RCC) between the patients with APF and non-APF. *P* values <0.05 were considered statistically significant. All statistical analyses were performed using SPSS version 20.0.

## 3. Results

### 3.1. Patients' Clinical and Radiographic Characteristics

The clinical and radiographic characteristics in our study are given in Table 1. The PFAD was graded as none, mild, moderate, and severe in 23.8%, 33.3%, 21.4%, and 21.4% of our patient cohort, respectively. Of these, 57% of the patients were over 55 years. The majority of patients were male (61.9%) and had malignant tumors (61.9%). The proportion of patients with malignant tumors and hypertension increases with the PFAD, as well as the median perinephric fat area and perinephric fat thickness. Only 2 patients had a history of chronic nephritis and both were in the SPF group. Proportion of patients with none, mild-to-moderate, and severe perinephric stranding were 61.9%, 28.6%, and 9.5%, respectively.

### 3.2. Clinical Characteristics for PFAD

Table 2 provides the clinical characteristics of PFAD, and bold values indicate significantly correlated factors. PFAD was significantly associated with clinical parameters including increasing age (OR 3.76, *p* = 0.003), male gender (OR 13.14, *p* < 0.001), and hypertension (OR 3.28, *p* = 0.006) on multivariable analysis, whereas other clinical variables were not associated with PFAD.

### 3.3. Radiological Predictors for PFAD

As given in Table 3, all of radiological variables were found to be predictive of PFAD (all *p* < 0.001, marked as bold values) on univariable analysis. However, multivariate analysis showed that perinephric stranding (type 1 OR 25.05, *p* = 0.001; type 2 OR 35.21, *p* = 0.033), posterior fat thickness (OR 11.46, *p* = 0.009), and perinephric fat density (OR 1.08, *p* = 0.02) appeared to be the most predictive of PFAD.

### 3.4. Impact of APF on Perioperative Outcomes and Pathological Grading

The 84 cases comprised 36 with APF (MoPF or SPF) and 48 with non-APF (NPF or MiPF). Drain output is significantly higher in the APF group (169 vs. 125.5 ml, *p* = 0.012, marked as bold values; Table 4). Other intraoperative variables (OT, WIT, EBL, and transfusions), postoperative variables (postoperative gastrointestinal recovery time, length of stay, postoperative fever, postoperative gastrointestinal discomfort, change dressing frequency, and CDC), and Fuhrman grading were similar between the two groups (*p* > 0.05).

### 3.5. Histological Analysis

**Figure 3:**
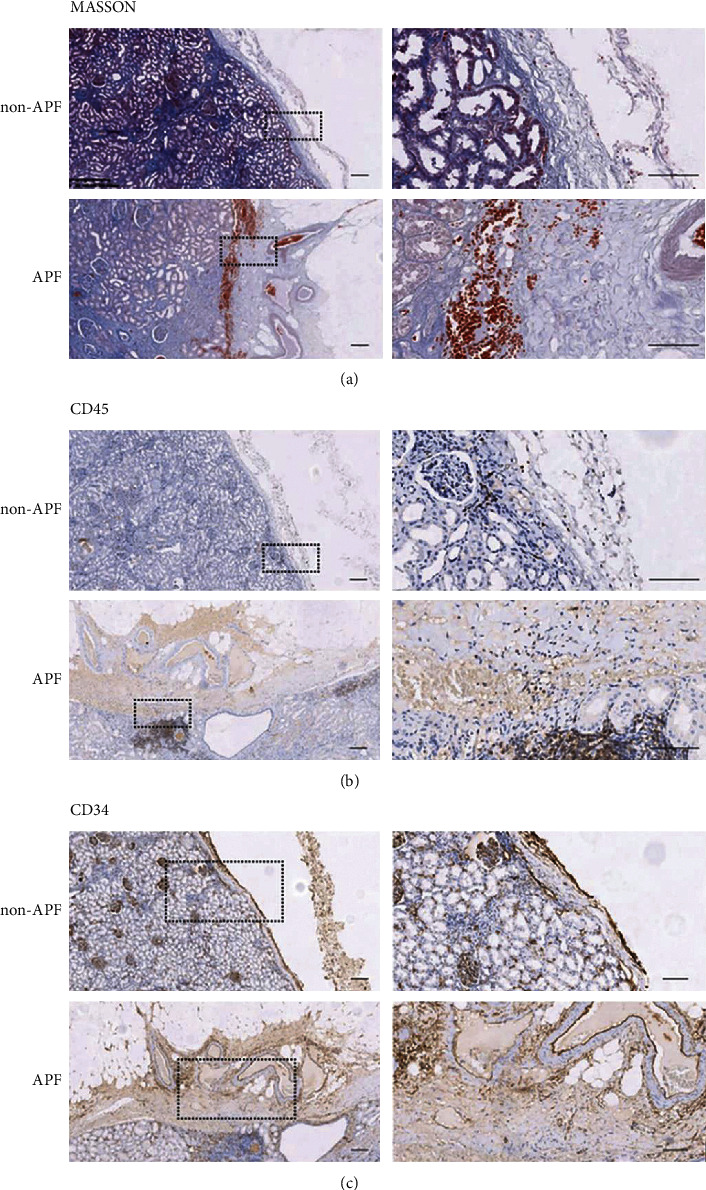
Representative examples of Masson, CD45, and CD34 staining. (a) Non-APF group having a thin extracapsular fascia, APF group renal capsule, and extracapsular fascia fusion thickening. (b) APF CD45 immune cells in the group highly expressed in the renal capsule and adjacent renal cortex. (c) APF group CD34 for vascular staining. Scale bar = 100 mm.

Masson staining showed that renal capsule was thin and had two layers in the non-APF group. Our study defined the outer membrane-like structure as extracapsular fascia. Patients with APF lack extracapsular fascia and the thickness of the renal capsule increased significantly (Figure 3(a)). Immunohistochemistry showed that CD45+ immune cells accumulate in the renal cortex and infiltrate into renal capsule in the APF group (Figure 3(b)). In patients with APF, the CD34+ vascular endothelial cells were significantly increased and arranged into a vascular-like structure (Figure 3(c)).

## 4. Discussion

APF is one of potential tumor-specific factors that can complicate partial nephrectomy (PN) and is associated with decreased progression-free survival in patients with localized renal cancer [6, 10, 14–16]. Previous studies on the association of APF with clinical, radiological features, or perioperative outcomes are given in Table 5. Due to lack of objective criteria for APF, the incidence of APF varies greatly. Therefore, our study established objective criteria after multiple surgical videos were reviewed. In this study, the incidence of APF was 42.8%, which was between the documented incidence [6, 8, 10].

Most literatures have confirmed that APF is associated with advanced age and common in males [4–6, 8–10, 14], which corresponds with our study. Male patients have more perinephric fat, whereas women have more subcutaneous fat [12, 17]. However, in the age, adipose tissue redistributes from subcutaneous to visceral and ectopic fat, especially along the kidneys, liver, and bone marrow. Adipose tissue is an endocrine organ which produces hormones such as cytokines especially in tumor necrosis factor-*α* (TNF*α*) and interleukin (IL-6). They can increase with aging and excessive accumulation of fat [18]. It was confirmed that the APF group solely increased the expression of sIL-6R, suggesting that APF may be a pathological procedure caused by systemic chronic inflammation [11, 19–21]. Therefore, both age and gender can affect the distribution of visceral and subcutaneous fat and also be the risk factors of APF.

In addition, our study also confirmed hypertension is the risk factor of PFAD, as previous studies reported [22, 23]. The immune system plays an important role in the development of hypertension, and renal immune cell infiltration has been demonstrated in both experimental and clinical hypertension [24]. In the SPF group, we first found that a large number of CD45+ immune cells accumulate in the renal cortex near renal capsule. There are also scattered immune cells, increased blood vessel distribution, and thickening of the renal capsule near renal cortex. Hypertension may affect the adhesion of the renal capsule to the perinephric fat by inducing renal immune cell infiltration. Two patients with previous nephritis were in the SPF group, adding weight to the theory that chronic inflammation of the kidney can affect fat adhesion [11, 25, 26].

Perinephric stranding and thickness of posterior perinephric fat are important radiological predictors for APF in this study. Perinephric stranding represents a chronic inflammatory reaction which is considered as an important factor for formation of APF. The posterior perinephric fat thickness represents excessive and dysfunctional adipose tissue. Based on the two variables, Mayo adhesive probability (MAP) score was established to predict APF [5], which has been validated in different surgical methods for renal cancer [6, 8, 10, 14, 23].

However, the predictive value for perinephric fat density is still controversial. Bylund et al. [4] found that the renal hilum level fat density had no significant effect on APF. Zheng et al. [9] pointed out that perinephric fat surface density can predict the difficulty of renal peritoneal fat separation. The above differences may be related to the measurement method of perinephric fat density. During surgery, we found that perinephric fat only adhered to the surface of the kidney and not to the posterior peritoneum or posterior abdominal wall, suggesting that the adhesion or inflammatory area may be closed to the kidney surface. So, we measure perinephric fat density in a high-density area adjacent to the renal capsule, and we found that some patients with SPF had significantly increased perinephric fat HU value on enhanced CT. Our results suggest that perinephric fat density may be a complement to the MAP score.

Several authors considered that APF can increase the risk of OT and bleeding during PN [6, 10, 14]. However, our results only find that drain output was associated with APF [27]. The OT and intraoperative EBL may be more affected by surgeon's surgical experience and other reasons, such as location of the tumor, damage of variant renal blood vessels and adjacent organs, and suture cutting off the renal parenchyma. Senior surgeons can speed up the surgical process and reduce damage and intraoperative bleeding, suggesting that whether APF can affect the OT and EBL needs further verification [28].

In this study, we found another new layer of fascia on the lateral side of renal capsule in the NPF group for the first time. This fascia was defined as extracapsular fascia. During PN, perinephric fat can be easily blunt dissected along the gap between renal capsule and extracapsular fascia. However, due to chronic inflammation for the SPF group, extracapsular fascia was taken place by thickening and fusion of renal capsule with many vessels, which will cause decapsulation and increasing hemorrhage in the process of kidney mobilization.

In conclusion, APF is more prevalent in aging and male populations, particularly in those with hypertension. Our research confirmed that APF is associated with drain output and accompanied by immune cells gathering in renal cortex near thickened renal capsule which with many vessels. Radiological factors show that perinephric stranding, posterior perinephric fat thickness, and perinephric fat density can be used to predict PFAD.

## Figures and Tables

**Table 1:** Clinical and radiographic characteristics stratified by adhesiveness of perinephric fat.

Characteristics	NPF (*n* = 20)	MiPF (*n* = 28)	MoPF (*n* = 18)	SPF (*n* = 18)	Total (*n* = 84)
Age (y)					
≤55	12 (60)	13 (46)	7 (39)	4 (22)	36 (43)
>55	8 (40)	15 (54)	11 (61)	14 (78)	48 (57)
Male gender (%)	6 (30)	14 (50)	15 (83.3)	17 (94.4)	52 (61.9)
BMI, kg/m^2^ (%)					
≤25	8 (40)	15 (53.6)	6 (33.3)	4 (22.2)	33 (39.3)
25–30	10 (50)	9 (32.1)	10 (55.6)	9 (50)	38 (45.2)
≥30	2 (10)	4 (14.3)	2 (11.1)	5 (27.8)	13 (15.5)
Diabetes (%)	5 (25)	6 (21.4)	4 (22.2)	6 (33.3)	19 (22.6)
Hypertension (%)	5 (25)	12 (42.9)	9 (50)	12 (66.7)	38 (45.2)
Cardiovascular disease (%)	3 (15)	2 (7.1)	3 (16.7)	2 (11.1)	10 (11.9)
Preoperative eGFR (<60 ml/min) (%)	0 (0)	0 (0)	0 (0)	2 (11.1)	2 (2.4)
Active smoking (%)	7 (35)	9 (32.1)	7 (38.9)	8 (44.4)	31 (36.9)
Alcoholism (%)	7 (35)	11 (39.3)	6 (33.3)	8 (44.4)	32 (38.1)
Malignancy (%)	10 (50)	16 (57.1)	12 (66.7)	14 (77.8)	52 (61.9)
Preoperative creatinine (>ULN) (%)	4 (20)	3 (10.7)	3 (16.7)	4 (22.2)	14 (16.7)
HU of perinephric fat	−103 (−106.6, −99.3)	−101 (−107.8, −96.3)	−95 (−106.6, −94.2)	−88 (−98.1, −81.5)	−98 (−106.5, −93)
Perinephric fat area (cm^2^)	0.69 (0.5, 1.3)	2.81 (1.8, 3.3)	5.44 (4.5, 7.1)	8.61 (6.2, 12.0)	3.05 (1.6, 6.2)
Perinephric fat thickness (mm)					
Medial	1 (0, 3.5)	5.9 (3.8, 7.7)	7.5 (4.9, 10.6)	9.6 (6.8, 17.2)	5.8 (3.1, 9.2)
Lateral	1.1 (0, 6.3)	10.1 (4.9, 15.0)	17.5 (12.2, 23.8)	22.5 (8.9, 25.3)	11.2 (4.3, 20.1)
Posterior	3.5 (2.3, 5.3)	7.8 (6.0, 11.8)	12.7 (8.7, 16.1)	19.4 (16.2, 31.4)	9.7 (5.7, 15.8)
Posterolateral	8.8 (5.7, 11.8)	13.8 (9.0, 16.9)	15.7 (14.0, 20.8)	24 (19.0, 29.6)	15.1 (10.2, 22.3)
Stranding (%)					
None	20 (100)	26 (92.8)	6 (33.3)	0 (0)	52 (61.9)
Type 1	0 (0)	2 (7.2)	11 (61.1)	11 (61.1)	24 (28.6)
Type 2	0 (0)	0 (0)	1 (5.6)	7 (39.9)	52 (9.5)

**Table 2:** Univariate and multivariable analyses clinical characteristics of PFAD.

Characteristics	Univariate		Multivariate	
	OR	*P* value	OR	*P* value
Age	2.61 (1.17, 5.82)	**0.019**	3.76 (1.57, 8.97)	**0.003**
Male gender	8.39 (3.32, 21.24)	**<0.001**	13.14 (4.87, 35.46)	**<0.001**
BMI				
≥30	2.65 (0.82, 8.54)	0.103		
25–30	1.58 (0.68, 3.68)	0.375		
Diabetes	1.83 (0.73, 4.62)	0.200		
Hypertension	2.85 (1.28, 6.33)	**0.010**	3.28 (1.40, 7.65)	**0.006**
Cardiovascular disease	0.99 (0.30, 3.25)	0.988		
Active smoking	1.34 (0.60, 298)	0.475		
Alcoholism	1.18 (0.53, 2.60)	0.685		
Malignancy	2.14 (0.95, 4.80)	0.065		
Preoperative creatinine	0.84 (0.30, 2.37)	0.747		

Bold values indicate significantly correlated factors.

**Table 3:** Univariate and multivariable analyses of predicting factors of PFAD.

Characteristics	Univariate		Multivariate	
	OR	*P* value	OR	*P* value
Perinephric fat thickness				
Medial	12.48 (4.79, 32.55)	**<0.001**		
Lateral	3.18 (2.01, 5.04)	**<0.001**		
Posterior	31.09 (10.62, 91.02)	**<0.001**	11.46 (1.86, 70.60)	**0.009**
Posterolateral	6.68 (3.43, 13.01)	**<0.001**		
Perinephric fat area	1.91 (1.55, 2.35)	**<0.001**		
HU of perinephric fat	1.14 (1.08, 1.20)	**<0.001**	1.08 (1.01, 1.14)	**0.020**
Stranding				
Type 1	113.44 (21.08, 610.32)	**<0.001**	25.05 (3.76, 167.00)	**0.001**
Type 2	986.26 (68.87, 14123.33)	**<0.001**	35.21 (1.32, 937.66)	**0.033**

Bold values indicate significantly correlated factors.

**Table 4:** Correlation between APF and perioperative outcomes and pathological grading of RCC.

Variable	APF	Control group with no APF	*P* value
Operative time (OT) (min)	110.50 (70.8, 126.0)	89 (85.3, 141.5)	0.141
Warm ischemia time (WIT) (min)	20.50 (17.8, 32.0)	21 (18.8, 25.3)	0.986
Drain output (ml)	169 (121.8, 186.3)	125.5 (94.5, 155.0)	**0.012**
Postoperative gastrointestinal recovery time (days)	5 (2.8, 6.0)	4 (3.0, 5.0)	0.611
Length of stay (days)	15 (13.8, 18.3)	14 (12.8, 15.3)	0.091
Transfusions	2 (5.6%)	3 (6.3%)	0.894
Estimated blood loss (EBL) (ml)	240 (195.5, 282.0)	215 (171.5, 270.8)	0.249
Postoperative fever (>38.0°C)	1 (2.8%)	4 (8.3%)	0.287
Postoperative gastrointestine discomfort	11 (30.6%)	10 (20.8%)	0.309
Change dressing frequency	4.5 (3.0, 6.3)	3 (2.0, 5.0)	0.052
Clavien–Dindo classification (CDC)	18 (50%)	18 (37.5%)	0.265
Clavien 1	15 (41.7%)	15 (31.3%)	
Clavien 2	3 (8.3%)	3 (6.3%)	
Fuhrman grade	26 (72%)	26 (54%)	0.106
I	10 (27.8%)	10 (20.8%)	
II	10 (27.8%)	11 (22.9%)	
III	2 (5.6%)	4 (8.3%)	
IV	4 (11.1%)	1 (2.1%)	

Bold values indicate significant difference.

**Table 5:** Summary of previous studies on APF.

Author (year)	Patient	Surgery	APF grading	APF rate (%)	Included factors	Significant variables
Bylund et al. (2013)	29	RPN, OPN, or laparoscopic cryoablation	Operative records	55.2	Clinical, imaging, pathological, outcome	Male gender, tumor size, stranding, tumor >50% exophytic, thickness of perinephric fat, and OT
Zheng et al. (2014)	41	OPN	Time of perinephric fat dissection on	53.7	Clinical, imaging, pathological, outcome	Male gender and PnFSD
Davidiuk et al. (2014)	100	RPN	Described by Kim et al.	30	Clinical, imaging, pathological, outcome	Male gender, BMI, posterolateral and posterior perinephric fat, and stranding
Davidiuk et al. (2015)	100	RPN	Described by Kim et al.	30	Outcome	
Kobayashi et al. (2016)	47	LPN or RALPN	Operative records	14.9	Clinical, imaging, outcome	OT, hypertension, and FSPA on CT
Martin et al. (2016)	86	OPN	Operative records	50.0	Clinical, imaging, outcome	Age and MAP score
Kocher et al. (2016)	245	LPN or RPN	Operative records	10.6	Clinical, imaging, pathological, outcome	Age, male gender, stranding, posterior fat thickness, MAP score, malignant renal histology, operating time, and EBL
Dariane et al. (2016)	125	RPN or OPN	Described by Kim et al.	40.8	Clinical, imaging, pathological, outcome, histological	OT, EBL, male gender, age, waist circumference, fat density on CT, MAP score, and larger adipocytes
Shintaro et al. (2017)	92	Laparoscopic donor nephrectomy	Intraoperative videos	55.4	Clinical, imaging, outcome, IHC	Perinephric fat area, stranding, sIL-6R, and OT
Khene et al. (2017)	202	RPN	Operative records	39.6	Clinical, imaging, outcome	Male gender, obesity, hypertension, MAP score, OT, EBL, transfusion, and conversion to open surgery or radical nephrectomy

## Data Availability

No data were used to support this study.

## Supplementary Materials

Supplementary Materials Representative examples of CD34 and CD45 immunohistological staining. (a) APF CD34 for vascular staining, positive in glomerular while negative in kidney tubules. (b) APF CD45 immune cells in the group, positive in lymph nodes while negative in normal kidney tissues. Scale bar = 80 mm.
