# Supplementary figures and images for: Analysis of Clinical Characteristics, Radiological Predictors, Pathological Features, and Perioperative Outcomes Associated with Perinephric Fat Adhesion Degree
Source: J Oncol. 2021 Dec 27;2021:9095469. doi: 10.1155/2021/9095469 (PMC8723850; doi:10.1155/2021/9095469)

**Supplementary materials**

**
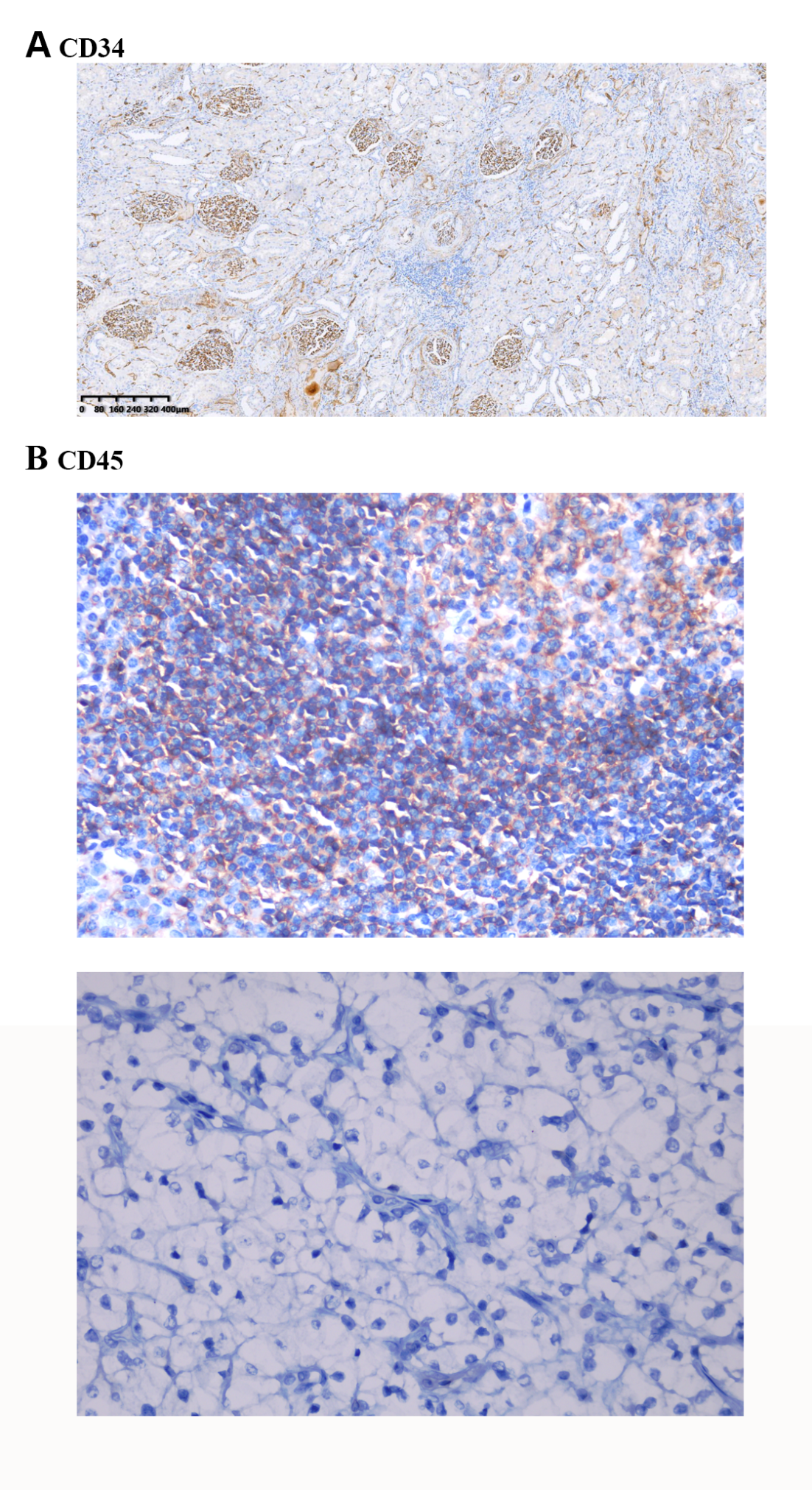
**

Supplement: Supplementary Materials — Representative examples of CD34 and CD45 immunohistological staining. (a) APF CD34 for vascular staining, positive in glomerular while negative in kidney tubules. (b) APF CD45 immune cells in the group, positive in lymph nodes while negative in normal kidney tissues. Scale bar = 80 mm. [file 9095469.f1.docx]
